# Adrenal Crisis in an Adult Patient With Lipoid Congenital Adrenal Hyperplasia due to STAR Protein Mutation

**DOI:** 10.1016/j.aed.2026.04.016

**Published:** 2026-05-05

**Authors:** Zhanna Zavgorodneva, Marija Kaljevic, Karin Chen

**Affiliations:** 1 Department of Endocrinology, SUNY Downstate Health Science University, Brooklyn, New York; 2 Division of Endocrinology, Department of Medicine, Maimonides Medical Center, Brooklyn, New York

**Keywords:** lipoid congenital adrenal hyperplasia, STAR protein, congenital adrenal hyperplasia, sex reversal

## Abstract

**Background/Objective:**

Congenital adrenal hyperplasia (CAH) comprises inherited disorders of adrenal steroidogenesis. Lipoid congenital adrenal hyperplasia (LCAH), caused by steroidogenic acute regulatory protein deficiency, is a rare and severe form characterized by complete or near-complete impairment of adrenal and gonadal steroid production. We report a case of adrenal crisis in an adult patient with LCAH.

**Case Presentation:**

A 22-year-old Yemeni woman with LCAH due to a homozygous steroidogenic acute regulatory mutation (exon 5, c.670C>T; p.A182C) presented with paresthesia and mild hypotension and was found to have significant hyperkalemia. Adrenal crisis was diagnosed, and she was treated with stress-dose glucocorticoids, resulting in resolution of symptoms and electrolyte abnormalities. Her medical history was notable for 46,XY sex reversal syndrome with primary amenorrhea, absent uterus, cryptorchidism requiring bilateral gonadectomy in childhood, and lifelong glucocorticoid and mineralocorticoid replacement, with estrogen therapy initiated at puberty. She also had hypothyroidism and a fixed Chiari I malformation. Despite long-term endocrinology follow-up, she had multiple prior admissions for salt-wasting crises with markedly elevated adrenocorticotropic hormone and plasma renin activity.

**Discussion:**

LCAH results in profound deficiency of mineralocorticoids, glucocorticoids, and sex steroids due to global impairment of steroidogenesis. Unlike 21-hydroxylase deficiency, LCAH is characterized by absent androgen production and lack of virilization in 46,XY individuals.

**Conclusions:**

Patients with severe forms of CAH may require higher glucocorticoid doses to prevent adrenal crises; however, overtreatment increases the risk of metabolic complications. Careful individualized titration of replacement therapy is essential.

**Highlights**

• Lipoid congenital adrenal hyperplasia (LCAH) is a rare and severe form of CAH characterized by near-complete loss of adrenal steroidogenesis
• Only a limited number of adult patients with LCAH have been described in the literature
• Among CAH subtypes, LCAH may carry a particularly high risk of adrenal crisis due to profound glucocorticoid and mineralocorticoid deficiency
• Patients with LCAH may require relatively higher glucocorticoid doses to prevent recurrent adrenal crises, necessitating careful long-term monitoring

**Clinical Relevance**

Genetic forms of congenital adrenal hyperplasia (CAH) are relatively uncommon in adult endocrine practice. When CAH presents in adulthood, it is most frequently attributable to 21-hydroxylase deficiency.

Congenital adrenal hyperplasia caused by mutations in the steroidogenic acute regulatory gene—also known as lipoid CAH—poses distinct diagnostic and therapeutic challenges. Clinicians must consider important management questions, including optimal glucocorticoid dosing strategies, prevention of recurrent adrenal crises, anticipated hospitalization risk, and recognition of the characteristic hormonal profile associated with this rare but severe form of CAH.

## Introduction

Adrenal crisis is a life-threatening emergency representing acute decompensation of adrenal insufficiency. Primary adrenal insufficiency is associated with more profound electrolyte disturbances and hemodynamic instability than secondary forms. Among the etiologies of primary adrenal insufficiency, congenital adrenal hyperplasia (CAH) comprises a distinct group of inherited disorders caused by defects in adrenal steroidogenesis. However, in adult endocrinology practice, adrenal crises more commonly result from autoimmune or infiltrative causes;^1^ CAH-related crises are encountered less frequently.

Adrenal crisis may occur despite appropriate glucocorticoid replacement and is often precipitated by infection, dehydration, surgery, emotional stress, or strenuous physical activity. Infections —particularly gastrointestinal illnesses—are the most common triggers.^2^ The reported incidence of adrenal crisis in patients with CAH is approximately 4.9-5.7 episodes per 100 patient-years,^2^ and adrenal crisis remains a leading cause of mortality in this population.^3^

The clinical severity and risk of adrenal crisis in CAH vary according to the underlying enzymatic defect. Classic 21-hydroxylase deficiency accounts for approximately 95% of cases and carries the highest risk of cortisol deficiency in the pediatric population.^4^^,^^5^ Other forms, such as 11β-hydroxylase deficiency,^6^ may impair cortisol synthesis while leading to excess androgen production due to precursor accumulation. In contrast, CAH caused by mutations in the steroidogenic acute regulatory (STAR) gene—also known as lipoid congenital adrenal hyperplasia (LCAH)—is a rare and severe variant characterized by complete (classical form) or near-complete (nonclassical form) loss of glucocorticoid, mineralocorticoid, and sex steroid synthesis.^6^^,^^7^

We report a case of adrenal crisis in an adult patient with genetically confirmed STAR-related CAH, highlighting this rare etiology and the persistent risk of recurrent adrenal decompensation, even after minor stressors, despite chronic high-dose steroid replacement.

## Case Presentation

A 22-year-old Yemeni woman with LCAH due to a homozygous STAR mutation (exon 5, c.670C>T; p.A182C), hypothyroidism, and fixed Chiari I malformation presented with 3 days of bilateral lower extremity cramps and paresthesia.

The patient was diagnosed with LCAH at 1.5 months of age following an adrenal crisis and had since been managed by the same pediatric endocrinology team with glucocorticoid replacement (hydrocortisone 30-40 mg a day over the last few years, most recently hydrocortisone 20 mg twice daily), mineralocorticoid replacement (fludrocortisone 0.3 mg twice daily), gonadal hormone replacement (transdermal estradiol 0.1 mg/d) and thyroid hormone replacement (levothyroxine 125 μg once daily). The patient reported adherence to her medications. She had a 46,XY disorder of sex development with phenotypic features consistent with LCAH, including female external genitalia, primary amenorrhea, and cryptorchidism requiring bilateral gonadectomy in childhood. Imaging studies demonstrated absence of the uterus and gonads.

Review of prior medical records revealed multiple hospitalizations for salt-wasting crises with persistently elevated adrenocorticotropic hormone (ACTH) levels ranging from 132 to 464.2 pmol/L (reference range: 1.3-16.7 pmol/L) and plasma renin activity ranging from 18.06 to 47.93 ng/mL/h (reference range: 0.25-5.28 ng/mL/h) (Table 1).

**Table 1:** Hormonal Profile Before and During Hospitalization

Date of the test	Cortisol AM (nmol/L)	ACTH (pmol/L)	PRA (ng/mL/h)	Estradiol (pmol/L)
During admission, after stress dose steroids	820	151		
04/17/2025	97	353	24.5	79
07/10/2024		464	47.93	
03/05/2024		299	18.06	
11/03/2021				74
11/16/2020		190	22.78	<2

Abbreviations: ACTH, adrenocorticotrophic hormone; PRA, plasma renin activity.

Reference range 2-12 pmol/L; Cortisol AM – 8 am Cortisol, reference ranges 138-690 nmol/L; reference ranges 0.25-5.28 ng/mL/h; Estradiol, reference range 70-1300 pmol/L depending on menstrual phase.

Three days prior to admission, while traveling, she developed progressive leg cramps and hand numbness, along with one episode of vomiting after a restaurant meal. Despite these symptoms, she continued taking her medications and doubled her glucocorticoid dose on the day of presentation according to prior sick-day instructions.

On presentation, the patient was alert and oriented without altered mental status. Initial blood pressure was 88/62 mm Hg and improved after administration of intravenous fluids and hydrocortisone 100 mg intravenously. All other vital signs were within normal limits.

Physical examination by the endocrinology team revealed mild macrocephaly, diffuse hyperpigmentation, and diminished bilateral knee reflexes. Secondary sexual characteristics were normal, with Tanner stage V breast development. Growth parameters were within normal limits without intellectual impairment or Cushingoid features, although she met criteria for class I obesity (weight 201 lb, height 5.38 ft, BMI 34).

Laboratory evaluation revealed hyponatremia, hyperkalemia, and a nonanion gap metabolic acidosis (Table 2). Electrocardiogram demonstrated normal sinus rhythm with tall T waves and a shortened QT interval of 329 ms, findings consistent with hyperkalemia.

**Table 2:** Laboratory Parameters During Hospitalization

Day of hospitalization	Potassium, mmol/L	Sodium, mmol/L	Bicarbonates, mmol/L	Cortisol, nmol/L
Day 1 of hospitalization, on admission	7.8	124	18	819
Day 2 of hospitalization	6.1	132	23	
Day 5 of hospitalization, on discharge	4.8	135	25	

Potassium, mmol/L (reference range 3.5-5.5); sodium, mmol/L (reference range 135-145); bicarbonate, mmol/L (reference range 22-28); serum cortisol, which was done after stress dose of steroids, nmol/L (reference range 138-635) are summarized in the table.

The patient was treated with sodium zirconium cyclosilicate 10 mg, intravenous insulin with dextrose, and nebulized salbutamol. Serum potassium improved to 6.3 mmol/L following initial therapy. Stress-dose glucocorticoids were continued with hydrocortisone 50 mg intravenously 3 times daily, followed by gradual tapering during hospitalization and transition to oral hydrocortisone. Fludrocortisone and levothyroxine were continued. Electrolyte abnormalities and symptoms resolved by hospital day 4.

The patient was discharged on her baseline regimen. At 3-week follow-up, she remained clinically stable with negative orthostatic vital signs and normal electrolyte levels.

## Discussion

The episode of adrenal insufficiency in our patient was promptly recognized and managed with standard glucocorticoid and mineralocorticoid replacement, requiring only brief hospitalization. Her symptoms resolved completely, and electrolyte abnormalities normalized prior to discharge. However, we would like to emphasize that the underlying etiology—LCAH due to a STAR gene mutation—is exceptionally rare. Approximately 250 cases have been reported worldwide,^8^ with around 50 occurring in adults. This case underscores the occurrence of adrenal crisis in an adult patient with this severe form of CAH.

STAR protein, first characterized in the 1990s by Stocco and Clark,^9^^,^^10^ plays a critical role in steroidogenesis by mediating the transport of cholesterol from the outer to the inner mitochondrial membrane in adrenal glands and gonads. This process represents the rate-limiting and obligatory first step in steroid hormone biosynthesis. In contrast to more common forms of CAH—such as 21-hydroxylase, 11β-hydroxylase, or 17α-hydroxylase/17,20-lyase deficiencies—STAR deficiency results in a far more profound disruption of adrenal and gonadal steroidogenesis. The failure to convert cholesterol to pregnenolone, the precursor of all steroid hormones, leads to severe salt wasting and disorders of sex development.^11^ STAR deficiency appears to be relatively more prevalent in East Asian^12^ and Middle Eastern populations.^13^ Multiple STAR gene mutations have been identified, including truncating variants, structural protein alterations, and mutations impairing cholesterol binding.^14^

A defining biochemical feature of STAR-related CAH is its distinctive steroid profile. Levels of 21-deoxysteroids—including 17-hydroxyprogesterone, typically elevated in 21-hydroxylase and 11β-hydroxylase deficiencies—are low or normal in STAR deficiency, making postnatal diagnosis more challenging.^8^^,^^11^ Unlike 3β-hydroxysteroid dehydrogenase deficiency, STAR deficiency does not lead to accumulation of 17-hydroxypregnenolone. Instead, there is near-complete loss of mineralocorticoid, glucocorticoid, and sex-steroid synthesis, reflecting global impairment of adrenal and gonadal steroidogenesis, which may create particular vulnerability to adrenal crisis. In contrast to 21-hydroxylase deficiency, patients with LCAH lack virilization due to absent androgen production, resulting in complete sex reversal in 46,XY individuals.^1^^,^^11^

In a comparative study of CAH subtypes, Koh et al^15^ identified overlapping clinical features between 21-hydroxylase deficiency and LCAH, including hyperpigmentation and susceptibility to early adrenal crisis. Several biochemical parameters—such as plasma renin activity, cortisol, and aldosterone levels—were also similar. However, ACTH concentrations were generally higher in LCAH. Importantly, unlike classic 21-hydroxylase deficiency, patients with LCAH tend to demonstrate slower bone-age advancement. This observation is clinically relevant, as there are currently no established guidelines for osteoporosis screening in CAH overall or specifically in adults with lipoid CAH.

With timely initiation and appropriate maintenance of glucocorticoid and mineralocorticoid replacement therapy, long-term survival is expected, and the number of recognized STAR-related CAH cases will likely increase as genetic testing becomes more widely accessible. In affected 46,XX individuals, estrogen replacement therapy supports normal pubertal development and induction of menarche, and pregnancy may be achievable in some cases spontaneously or with assisted reproductive technologies.^16^, ^17^, ^18^ Most individuals with a 46,XY karyotype also require sex hormone replacement therapy to promote the development of secondary sexual characteristics and to preserve bone mineral density.

CAH due to STAR deficiency—characterized by near-complete absence of endogenous steroid production—represents one of the most severe and crisis-prone forms of primary adrenal insufficiency. Even minor physiological stressors, such as gastrointestinal illness, dehydration, emotional stress, or missed medication doses, may precipitate adrenal crisis and recurrent hospital admissions. In prospective studies of chronic primary adrenal insufficiency, poor adherence to glucocorticoid therapy, hyponatremia, and interruptions in hydrocortisone treatment were identified as strong predictors of adrenal-crisis–related mortality,^19^ while gastrointestinal infections and emotional stress were among the most frequent triggers. Given these risks, patients with STAR-related CAH must receive comprehensive education regarding strict adherence to steroid replacement therapy, appropriate use of “sick-day rules,” and the necessity of carrying an emergency injectable glucocorticoid for situations in which oral intake or absorption is compromised.

Across all forms of CAH, clinicians must remain vigilant for long-term comorbidities, including obesity, psychiatric disorders, and cognitive impairment,^20^ which may be influenced by chronic glucocorticoid exposure. In severe steroidogenic defects, higher glucocorticoid doses may be required to prevent adrenal crises; however, supraphysiologic dosing increases the risk of metabolic complications. In our patient, hydrocortisone doses of 30-40 mg/d were required despite persistently elevated ACTH levels. However, these findings cannot be generalized to all adults with LCAH, as disease severity varies based on specific STAR mutations and individual factors such as adherence, body surface area, and glucocorticoid requirements. Careful titration of glucocorticoid therapy is therefore essential to balance adequate adrenal replacement with minimization of long-term adverse effects, with dose optimization guided during follow-up once clinical stability is achieved.

## Conclusions

Patients with severe forms of LCAH require careful individualized glucocorticoid replacement. Emphasis on adherence to therapy, stress-dose education, and close follow-up is essential to prevent adrenal crises while minimizing the risk of overtreatment and associated metabolic complications.

## Informed Patient Consent for Publication

Signed informed consent was obtained directly from the patient.

## Disclosure

The authors have no conflicts of interest to disclose.
